# Embolization of unruptured wide-necked aneurysms at the MCA bifurcation using the Neuroform atlas stent-assisted coiling: a two-center retrospective study

**DOI:** 10.3389/fneur.2023.1199390

**Published:** 2023-08-16

**Authors:** Xuexian Zhang, Ruidong Wang, Yuhan Ding, Wei Li, Hong Ren, Jun Zhang

**Affiliations:** ^1^Department of Neurointervention, Jingmen People's Hospital, Jingmen, Hubei, China; ^2^Interventional Department, Qujing Second People's Hospital, Qujing, Yunnan, China; ^3^Department of Oncology, Jingmen Central Hospital, Jingmen, Hubei, China

**Keywords:** middle cerebral artery, bifurcation, Neuroform atlas stent, stent-assisted coiling, aneurysm

## Abstract

**Background:**

The management of middle cerebral artery (MCA) aneurysms remains a controversial topic, and MCA aneurysms have traditionally been treated primarily by surgical clipping. The Neuroform Atlas Stent™ (NAS, available from Stryker Neurovascular, Fremont, California) represents the latest generation of intracranial stents with improved stent delivery system capabilities.

**Objective:**

This study aims to investigate the safety, feasibility and efficacy exhibited by NAS in treating unruptured aneurysms at the MCA bifurcation.

**Methods:**

This was a two-center retrospective study involving 42 patients with unruptured wide-necked aneurysms (WNAs) of the MCA treated with the NAS from October 2020 to July 2022.

**Results:**

The stent was used to treat 42 cases of unruptured WNA at the MCA bifurcation. Endovascular treatment techniques had a 100% success rate. Immediate postoperative angiography found complete aneurysm occlusion in 34 patients (80.9%) (mRRC 1), neck remnant in 7 patients (16.7%) (mRRC 2), and residual aneurysm in 1 patient (2.4%) (mRRC 3). The thromboembolic complication rate was 2.4% (1/42). The follow-up period was 8.7 months on average (3–16 months). The last angiographic follow-up results revealed complete aneurysm occlusion in 39 patients (92.9%) (mRRC 1), neck remnant in 3 (7.1%) patients (mRRC 2), no aneurysm recanalization or recurrence, and no cases of stent intimal hyperplasia. During the latest clinical follow-up, all patients had an mRS score of 0.

**Conclusion:**

Our study demonstrates that the NAS can be applied to treat unruptured WNAs at the MCA bifurcation with favorable safety, feasibility, and efficacy.

## Introduction

Intracranial aneurysms are increasingly treated with endovascular approaches (1). Middle cerebral artery (MCA) aneurysms account for 18 to 36% of all types of intracranial aneurysms, the majority of which are located at the MCA bifurcation (2). The treatment of MCA aneurysms remains a controversial topic. The traditional therapeutic regimen for MCA aneurysms is surgical clipping. Despite this fact, since the publication of results from the International Subarachnoid Aneurysm Trial (ISAT) and the International Study of Unruptured Intracranial Aneurysms (ISUA) on the safety and efficacy exhibited by endovascular interventions, endovascular treatments for these aneurysms have been improved for wider implementation (3). Studies have confirmed the better performance of stent-assisted coiling (SAC) than that of single embolization for both ruptured and unruptured aneurysms (4). Due to the different geometries and configurations of aneurysms, conventional simple embolization can present several complications, including coils protruding from the aneurysm lumen or even dislocating into the parent artery. The above complications can easily develop in wide-necked aneurysms (WNAs), which are aneurysms possessing a neck diameter ≥ 4.0 mm or a dome-to-neck ratio < 2.0. SAC is considered to be one of the most important methods for treating WNAs; it can minimize the risk of protrusion or dislocation and can effectively and safely be applied to treat these intracranial aneurysms (5, 6). The latest generation Neuroform Atlas Stent (NAS, Stryker Neurovascular, Fremont, CA) represents the latest advancement in laser-cut microstents for the brain, a low-profile self-expanding stent possessing an open-hole design. It is used to support a removable intrasaccular coil, preventing the coil from protruding out of the aneurysm lumen while providing flow redirection with low shunt rates. The original Neuroform stent was first approved more than 10 years ago, and its safety and efficacy for assisting coil embolization of aneurysms has been proven many times around the world. Considering the extensive clinical experience regarding the first-generation Neuroform stent, the new-generation Neuroform stent aims to improve the delivery of microcatheters for coil embolization in the same way. Its inner diameter is 0.0165 inches (unlike the 0.027-inch Neuroform EZ) due to its smaller size, and consequently, it can be placed in more distal vessels, such as branches of the anterior and middle cerebral arteries. Additionally, new features add a hybrid cell design, with the Atlas stent partially deployed proximally and distally to provide excellent wall apposition, aneurysm neck coverage, and robust coil support. Three years ago, the FDA approved the Atlas stent for treating WNAs during the anterior circulation. The results from multiple previous studies using the NAS for SAC of intracranial aneurysms have increased our confidence in this device (6–9). Data on safety and long-term efficacy are sparse, and further research is needed. Therefore, the present study aims to report our experience in patients with unruptured aneurysms at the MCA bifurcation using NAS-assisted embolization.

### Methods

The retrospective multicenter study was approved by the hospital ethics committee, and the need for informed consent was waived.

### Patients

The study conducted a retrospective analysis of all patients exhibiting unruptured WNAs at the MCA bifurcation under treatment by the Neuroform Atlas device at two institutions between October 2020 and July 2022. The study included 42 patients with WNAs at the MCA bifurcation. Inclusion of these patients was determined by an interdisciplinary consensus panel based on our inclusion and exclusion criteria. The general data and clinical data of the patients were collected. The clinical data include the basic characteristics of aneurysm (aneurysm length, aneurysm neck size, aneurysm location), treatment results, and follow-up data.

### Inclusion and exclusion criteria

Inclusion criteria: (1) cranial MRA or head and neck CTA or DSA examination and confirmed unruptured aneurysm at the MCA bifurcation; (2) Refusal of surgical clipping requiring endovascular therapy; (3) adjuvant therapy with NAS when receiving endovascular therapy. Exclusion criteria: (1) dissecting aneurysm, pseudoaneurysm or ruptured aneurysm; (2) intracranial aneurysm complicated with other cerebrovascular diseases, such as intracranial arteriovenous malformation and moyamoya disease; (3) incomplete case data.

### Perioperative antiplatelet management

The patients started taking aspirin (100 mg/d) combined with clopidogrel (75 mg/d) antiplatelet therapy at least 7 days before treatment. We performed a platelet function test (TEG Hemostasis System, Hemoscope Corporation, Niles, IL, United States) one week after the dual antiplatelet drugs to evaluate the inhibitory effect of dual antiplatelet drugs on platelet aggregation. When the patient failed to exhibit sensitivity to clopidogrel, ticagrelor (90 mg twice daily) was used instead of clopidogrel. Patients again underwent dual antiplatelet therapy with aspirin and either clopidogrel or ticagrelor for no less than 3 months after endovascular therapy. Clopidogrel or ticagrelor was discontinued at 3 months, and the patient was continued on aspirin for life.

### Endovascular treatment procedures

After general anesthesia, all endovascular procedures were performed using Siemens Artis Zee angiography equipment. After successful femoral artery puncture, an 8F femoral artery sheath was placed, with intravenous infusion of heparin (50 U/kg) and an additional 1,000 U per hour to obtain an activated coagulation time between 2 and 3 of baseline in the process of operation 3 times. We placed the 6-F long sheath into the C1 segment of the internal carotid artery (ICA) through the 8F femoral artery sheath and the intermediate catheter into the C5 segment of the ICA through the long sheath. Based on the reconstructed images, an appropriate working projection was selected, namely, the angiographic projection that most clearly demonstrated the association of the aneurysm neck with the parent vessel. Angiographic evaluation assessed the aneurysm size and selected the appropriate coil based on the size of the aneurysm. Excelsior SL-10 microcatheters were used to deliver the NAS. It was at the operator’s discretion whether to create a “shelf” in a Y-bracket configuration for more neck coverage. One patient was treated with dual stents in the Y configuration. Considering the aneurysm neck size and the parent artery diameter, we selected a stent with an appropriate length and diameter for completely covering the aneurysm neck, and the “half-release” technique was used to release the stent while packing the coil. After the stent was completely released, stent deployment, stent adherence, stent patency, and tumor cavity filling were observed at the working angle and anterior and lateral angiography. The indwelling arterial sheath was pulled out 8 h after the operation. Immediately after the operation, a head CT scan was performed to observe whether there was intracranial hemorrhage. After recovery from anesthesia, the patients were returned to the ward. After discharge, patients were asked to return periodically for a review of their cerebral angiography and for clinical follow-up.

### Angiography and clinical follow-up

The modified Raymond Roy classification scale (mRRC) assisted in assessing the effectiveness of aneurysm embolization, with mRRC1, 2 and 3 representing complete aneurysm occlusion, neck remnant, and residual aneurysm, respectively (10). The patients were instructed to return to the hospital for DSA angiography at 3, 6, and 12 months after treatment. The following two conditions were defined as aneurysm recanalization: 1. conversion of an initial complete aneurysm occlusion to a residual neck or residual aneurysm; 2. conversion of an initial residual neck to a residual aneurysm. Two experienced neurointerventionalists reviewed all images and agreed on the angiographic findings. The modified Rankin Scale (mRS) was employed to assess the clinical outcomes of patients when they were discharged and during the most recent follow-up.

### Statistical analysis

SPSS 25.0 statistical software was used for the data analysis. This single-arm study only investigated the outcomes of NAS-assisted embolization in treating unruptured WNAs at the MCA bifurcation and did not compare the outcomes with those of other types of treatment; therefore, only descriptive statistics are provided. All data are presented as the means and ranges of continuous variables as well as the numbers and percentages of categorical variables.

## Results

### Baseline characteristics

Forty-two patients who received NAS for unruptured WNAs at the MCA bifurcation were included in our study, including 16 males and 26 females (mean age: 59.21 years; range 41–78 years). Hypertension was found in 25 cases (59.5%), diabetes in 5 cases (11.9%) and smoking in 11 cases (26.2%). Aneurysm was found on physical examination in 12 patients, headache in 14 patients, dizziness in 10 patients, and tinnitus in 6 patients. The aneurysm was located on the left side in 22 patients, and the aneurysm in the remaining 20 patients was located on the right side. The aneurysms possessed an average maximum diameter of 4.3 ± 1.0 mm (2.8–7.5 mm) and an average neck diameter of 2.8 ± 0.8 mm (1.8–6.4 mm). The mean aneurysm dome/neck ratio was 1.62 ± 0.2, and the average PHASES Score of the patients is 2.7 ± 0.6.Baseline data are shown in Table 1.

**Table 1:** Baseline and aneurysm characteristics of patients.

Variable value^a^	
Gender (n)	
Male	16 (38.1%)
Female	26 (61.9%)
Age (years)	59.21 ± 8.90
Clinical risk factors (*n*)	
Smoking	11 (26.2%)
hypertension	25 (59.5%)
diabetes	5 (11.9%)
Location	
Left	22 (52.4%)
Right	20 (47.6%)
Symptom	
Asymptomatic	12 (28.6%)
Headache	14 (33.3%)
Dizziness	10 (23.8%)
Tinnitus	6 (14.3%)
Aneurysm maximum diameter (mm)	4.3 ± 1.0
Aneurysm neck diameter (mm)	2.8 ± 0.8
Aneurysm dome/neck ratio	1.6 ± 0.2
PHASES Score	2.7 ± 0.6

a Values are mean ± standard deviation or number of patients (percentage).

### Immediate embolic effects and endovascular treatment-related complications

The endovascular treatment success rate was 100%, and there was no NAS placement failure. Immediate postoperative angiography revealed complete aneurysm occlusion in 34 patients (80.9%) after endovascular treatment (mRRC 1), 7 patients (16.7%) with neck remnant (mRRC 2), and 1 patient (2.4%) with residual aneurysm (mRRC 3) (Table 2). The thromboembolic complication rate was 2.4% (1/42). One patient developed thrombosis, which disappeared after to tirofiban bolus injection. The other patients did not experience thrombosis, postoperative intracranial hemorrhage or other surgery-related complications. The patients with intraoperative thrombosis had mild right extremity dysfunction at discharge with an mRS score of 1, and the rest possessed an mRS score of 0 when they were discharged (Table 2).

**Table 2:** Immediate and follow-up results.

Variable value^b^	
Immediate postoperative embolism classification	
mRRC 1	34 (80.9%)
mRRC 2	7 (16.7%)
mRRC 3	1 (2.4%)
mRS score at discharge	
0	41 (97.6%)
1	1 (2.4%)
2	0 (0%)
3	0 (0%)
4	0 (0%)
5	0 (0%)
6	0 (0%)
Mean follow-up time(months)	8.67 ± 2.98
Results of the last follow-up angiography	
mRRC 1	39 (92.9%)
mRRC 2	3 (7.1%)
mRRC 3	0 (0%)
mRS score at last clinical follow-up	
0	42 (100%)
1	0 (0%)
2	0 (0%)
3	0 (0%)
4	0 (0%)
5	0 (0%)
6	0 (0%)

b Values are mean ± standard deviation or number of patients (percentage). mRS, Modified Rankin Scale.

### Clinical and angiographic follow-up

**Figure 1:**
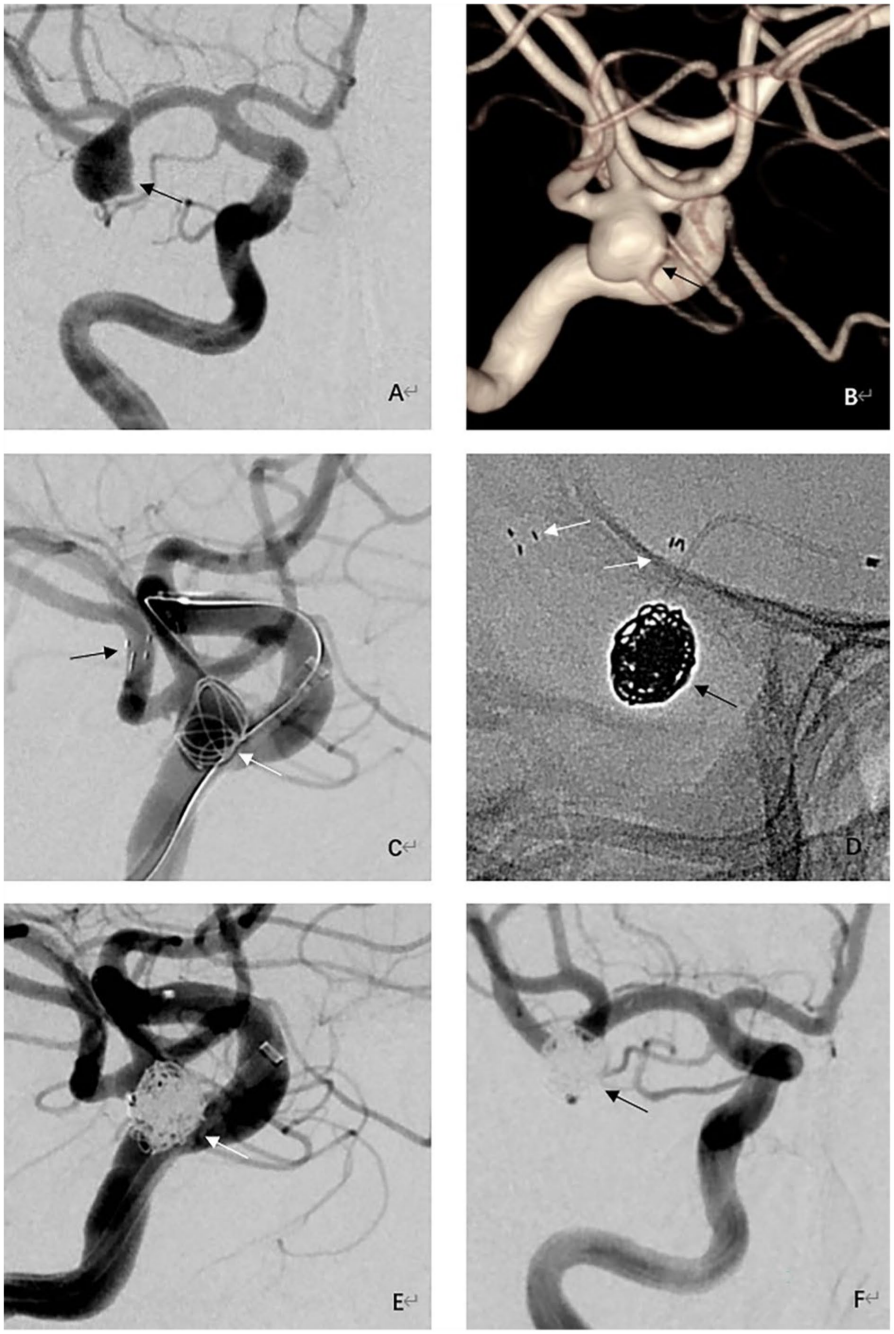
Procedural and follow-up angiographic images of an adult patient with an unruptured wide-necked aneurysm at the right MCA bifurcation. **(A)** Preprocedural right internal carotid angiography image showing a wide-necked aneurysm at the MCA bifurcation (black arrow). **(B)** Preprocedural 3-dimensional reconstructed angiography image showing a saccular wide-necked MCA aneurysm (black arrow). **(C)** Intraoperative angiographic image shows Neuroform Atlas stent (black arrows) deployed into the inferior trunks of the MCA and coils (white arrow) inside the aneurysm. **(D)** Visualization of stent (white arrows) and coils (black arrow) under fluoroscopy. **(E)** Immediate postprocedural angiographic image showing complete embolization of the aneurysm (white arrow). **(F)** 9-month follow-up angiography demonstrating complete occlusion of the aneurysm (black arrow) with good branch preservation and no in-stent stenosis.

Clinical and angiographic follow-up results were available for all 42 patients. The follow-up period lasted 8.7 months on average (3 to 16 months). The last angiographic follow-up revealed complete aneurysm occlusion in 39 patients (92.9%) (mRRC 1), neck remnant in 3 (7.1%) patients (mRRC 2), and no aneurysm recanalization or recurrence. The last angiographic follow-up showed no stent intimal hyperplasia in any patient (case illustration, Figure 1). None of the patients developed bleeding or thromboembolic events during clinical follow-up. At the most recent follow-up, the mRS score was 0 for all patients. The follow-up data are shown in Table 2.

## Discussion

Endovascular treatment is challenging for many MCA aneurysms, especially aneurysms at MCA bifurcations. Although many innovative devices have emerged over the past decade with the development of neurointerventional devices, many neurovascular centers still view microsurgical clipping as the treatment of choice (1). A more complex and controversial topic lies in the management of WNAs (neck ≥4 mm) and small aneurysms located distal to the branch of the MCA (round neck ratio < 2), which are often treated with clipping (3). Conventional coil embolization specific to wide-neck MCA bifurcated aneurysms, without the assistance of stents or balloons, remains a technical challenge due to the bifurcation anatomy, wide neck, deformed shape, and angiographically unrecognizable branches, and the stability of its long-term results remains controversial (11). Wide-neck intracranial aneurysms need additional devices like balloons or stent for management (12) Stent-assisted coiling achieved better results in terms of complete occlusion and stability than balloon-assisted coiling with a lower rate of recurrence without being associated with a higher risk of intraprocedural complications (13). Woven EndoBridge [WEB (Sequent Medical, Aliso Viejo, California, USA)] is a highly innovative technique for the endovascular treatment of wide-necked bifurcation aneurysms (WNBAs), but it is more suitable for treatment of basilar bifurcation aneurysms, WNBAs with necks >7 mm, and/or those with a bifurcation angle >180° (14). With the development of various neurointerventional devices and neurointerventional techniques, many studies have revealed the effectiveness and safety of stent-assisted embolization in treating MCA aneurysms. YAN et al. reported the results of 57 patients with unruptured WNAs at the MCA bifurcation under the treatment of Low-Profile Visualization Endovascular Support (LVIS) stent-assisted embolization (15). Immediate postoperative angiography showed that 45.6% of aneurysms had Raymond 1 occlusions (45.6%), 17.6% of the aneurysms had Raymond 2 occlusions, and two patients (3.5%) experienced perioperative complications, one with a procedure-related bleeding event and the other with a procedure-related thromboembolic event. Angiographic results obtained at an average follow-up of 11.7 months after surgery showed complete occlusion in 78.7%, improvement in 6.4%, stabilization in 10.6%, and recanalization in only 4.3% of cases. During follow-up, 1 in-stent stenosis was found, and 2 patients developed stagnation or occlusion of occluded branches, but none of the three patients developed symptoms. There were no thromboembolic or bleeding events in any patient in the clinical follow-up. LVIS stent-assisted embolization has shown good long-term outcomes. Hegan et al. retrospectively analyzed the outcomes of 150 unruptured MCA bifurcations treated with coils, SAC, or intravascular shunts (WEB devices), 45 of which were stent-assisted embolizations (16). The results of this study showed that the overall surgery-related good clinical outcomes (mRS ≤2) were found in 89.9% of patients, and the mortality was 2.7%; short-term follow-up was conducted for 91.3% of patients, and the mortality was 0.7%. Long-term angiographic follow-up showed stent-assisted embolization recurrence. The treatment rate was only 5.9%. This study demonstrates that endovascular treatment has good clinical outcomes and lower mortality regardless of the structure of the MCA bifurcation aneurysm. Yunsun et al. reported the results of 14 patients suffering MCA aneurysms undergoing stent-assisted embolization of acute hypoplastic M1 branches, and 13 patients achieved an average bulk density of 30% (17). Magnetic resonance angiography at an average follow-up of 4 months (1–26 months) revealed that 11 patients had developed complete occlusion, and 3 patients had developed a residual aneurysm neck. At a median clinical follow-up of 17 months (2–26 months), there were no clinical events, with an mRS score 0. One patient exhibited thrombotic occlusion during surgery that resolved with tirofiban infusion, with no evidence of infarction or defect. Chen et al. reported the results of SAC using the solitaire AB stent at the MCA trifurcation in 57 WNA patients (18). Immediate postoperative angiography showed complete embolization in 52 cases, residual aneurysm neck in 4 cases, and residual aneurysm body in 1 case. Fifty patients were followed for 6–36 months. No aneurysmal rupture or hemorrhage was observed during the clinical follow-up. According to the last angiographic evaluation, 46 patients developed complete embolization, 3 patients developed a residual neck, 1 patient developed an aneurysm body, and the other 2 patients developed a stable aneurysm. Finally, patients with residual aneurysm body were asymptomatic at follow-up review. Hidenori et al. reported the angiographic and clinical outcomes of SAC of 47 unruptured MCA aneurysms in 46 patients treated with Low Profile Visualization Endovascular Support Primary (LVIS Jr.) stents (19). Immediate postoperative angiographic findings were Raymond-Roy class I in 31 patients (65.0%), Raymond-Roy class II in 5 patients (10.6%), and Raymond-Roy class III in 11 patients (23.4%). According to the latest angiographic results, 33 cases (86.8%) were Raymond-Roy class I, 2 cases (5.3%) were Raymond-Roy class II and 3 cases (7.9%) were Raymond-Roy class III. The aneurysm occlusion status remained unchanged in 27 cases (71.0%), improved in 9 cases (23.7%), and worsened in 2 cases (5.3%). No recurrent aneurysm required additional treatment. Two patients had significant stent thrombosis on angiography, but their clinical outcomes remained favorable. At discharge, the mRS score was 0 in 45 patients and 1 in 1 patient. Feng et al. reported the safety and efficacy of LVIS Jr. stents used for MCA aneurysm embolization (20). There were 18 MCA aneurysms, with 13 unruptured and 5 ruptured aneurysms. At the last angiographic follow-up, 8 patients (44.4%) had complete occlusion, 7 patients (38.9%) had residual neck, and 3 patients (16.7%) had partial occlusion. One patient had intraoperative stent thrombosis, which disappeared due to intravenous injection of tirofiban. At discharge, 14 patients had mRS scores of 0; 3 patients, scores of 1; and 1 patient, a score of 2.

The past two decades saw the emergence of many newer laser-cut or braided stents, such as the Neuroform EZ, Solitaire, Enterprise, LVIS and LVIS Jr. stents. Each bracket possesses advantages and limitations. Compared with closed-cell stents, open-cell stents exhibit a strong vessel wall fit but cannot be recoated, and the coils are more likely to come out in patients using open-cell stents. Braided stents increase metal coverage for better shunting but are not easy to deploy (21). Embolization of MCA aneurysms is often challenging due to the wide neck anatomy associated with bifurcations. This forces the surgeon to often choose between coiling the tumor lumen and damaging the parent or distal vessels. To this end, stents specifically developed for SAC, including Y-stents and the NAS, largely solve this problem (9). The NAS (Stryker Neurovascular, Fremont, CA, United States) serves as a laser-cut, self-expanding nitinol stent that provides good adherence and sufficient linear support in stent-assisted embolization to prevent coil prolapse. The atlas possesses a hybrid cell structure consisting of alternating 16-pole and 24-pole rows. The design aims to enhance the adherence and compliance of the stent. In addition, the NAS features a minimal see-through design. It is available in 3.0, 4.0 and 4.5 mm diameters and four lengths: 15, 21, 24 and 30 mm; the bracket is designed to be deployed on female 2–3, 3–4 and 4–4.5 mm diameters, respectively, in blood vessels. Stent systems are delivered via microcatheters, including XT-17™ Microcatheters (0.017” ID) (Stryker Neurovascular) and Excelsior SL-10® (0.0165” ID). Its small inner diameter delivery system makes it capable of increasing the aneurysm types that can be treated within the vessel, such as those that are previously inaccessible (22). Zaidat et al. reported one-year follow-up results of the NAS for anterior circulation aneurysms, achieving complete occlusion in 88.2% of patients, parent artery stenosis (>50% stenosis) in 1.3% of patients, and 7 patients underwent retreatment, 84.7% of patients achieved complete aneurysm occlusion (Raymond-Roy class 1) with retreatment of the aneurysm or stenosis of the parentless artery, and 4.4% experienced ipsilateral stroke or neurological death (7). This study demonstrates that the NAS system can assist in safely and effectively treating WNAs of the anterior circulation. Kim et al. reported data on application of the NAS in patients with ruptured and unruptured cerebral aneurysms (21). Thirty-three aneurysms in 31 patients received embolization with the assistance of the NAS. Of the 29 aneurysms under angiography after patients were followed up for 6 months, 19 (65.5%) had Raymond-Roy class I occlusions, 8 (27.6%) had Raymond-Roy class II occlusions, and 2 (6.9%) had Raymond-Roy class III occlusions. No patients experienced surgery-related bleeding complications. Sweid et al. published a retrospective multicenter study of 69 subjects treated with the Atlas stent (23). At the 4-month follow-up, 97.7% of aneurysms had Raymond-Roy class I/II occlusion results, 91.8% of patients had good clinical outcomes, and the mortality rate was only 1.4%. A meta-analysis using NAS-assisted coiled intracranial aneurysms showed that the NAS-assisted coil achieved an initial adequate occlusion rate of 88% and the perioperative complication rate was 6% (5). The immediate postoperative complete occlusion rates were 85 and 86% for unruptured aneurysms and WNAs, respectively. Kubilay et al. illustrated the results of Y-stent-assisted coiling with NAS for treating WNAs with complex intracranial bifurcation. The study included 30 patients, and 30 aneurysms were treated postoperatively (8). Immediate angiography revealed complete aneurysm occlusion in 83.3% of patients. The angiographic follow-up lasted 11.8 months on average. The last follow-up revealed complete aneurysm occlusion in 93.3% of patients. Only 6.7% of patients developed surgery-related complications, 3.3% of patients developed permanent complications, and no patients died. Hanel et al. presented results from a prospective study trial of the NAS in treating MCA aneurysms (9). A total of 35 patients (27 MCA bifurcations, 5 M1, 3 M2) were included. Twenty-six patients received digital subtraction angiography at 12 months, of whom 80.8% (21/26) had complete aneurysm occlusion. At 1 year, 84.4% (27/32) of patients had an mRS score of ≤2, and 3 patients were lost to follow-up. Stent-assisted crimping with the NAS is an alternative to surgical clipping for selected MCA aneurysms.

A total of 42 patients with unruptured WNAs at the MCA bifurcation were included in the study. The follow-up lasted 8.7 months on average. The last angiographic follow-up revealed complete aneurysm occlusion in 92.9% of patients (mRRC 1), neck remnant in 7.1% of patients (mRRC2), no patients with aneurysm recanalization or recurrence, and no patients with stent intimal hyperplasia. For all patients, the mRS score was 0 at the last clinical follow-up. The present study shows favorable results for application of the NAS in unruptured aneurysms at the MCA bifurcation.

### Limitations

The present investigation has some limitations. First, it was a retrospective study with a small sample size, making the conclusions drawn less persuasive. Future studies with larger sample sizes are needed to better evaluate the safety and efficacy exhibited by the Atlas stent in treating unruptured WNAs at the MCA bifurcation. In addition, this was a multicenter study conducted in conjunction with another institution, and although uniform standards were used in data collection, interobserver and reporting biases may still have been introduced. In addition, our follow-up time was also shorter, which may not accurately reflect the aneurysm occlusion rate, requiring a longer follow-up time to draw more reliable conclusions.

## Conclusion

Although many challenges remain in treating unruptured WNAs located at MCA bifurcations to date, our study demonstrates that the NAS can safely and feasibly treat such lesions. Accordingly, it is clear that the NAS contributes greatly to the treatment of intracranial aneurysms, and we have immense confidence in this device.

## Data availability statement

The raw data supporting the conclusions of this article will be made available by the authors, without undue reservation.

## Ethics statement

The studies involving human participants were reviewed and approved by Jingmen People’s Hospital and Qujing Second People’s Hospital (2022-073-01). Written informed consent for participation was not required for this study in accordance with the national legislation and the institutional requirements.

## Author contributions

XZ, RW, and WL: designing or planning the manuscript. XZ and YD: drafting the manuscript. JZ: critically reviewing and editing. HR and WL: approving the final version. All authors contributed to the article and approved the submitted version.

## Conflict of interest

The authors declare that the research was conducted in the absence of any commercial or financial relationships that could be construed as a potential conflict of interest.

## Publisher’s note

All claims expressed in this article are solely those of the authors and do not necessarily represent those of their affiliated organizations, or those of the publisher, the editors and the reviewers. Any product that may be evaluated in this article, or claim that may be made by its manufacturer, is not guaranteed or endorsed by the publisher.
